# Non-Contact Monitoring and Classification of Breathing Pattern for the Supervision of People Infected by COVID-19

**DOI:** 10.3390/s21093172

**Published:** 2021-05-03

**Authors:** Ariana Tulus Purnomo, Ding-Bing Lin, Tjahjo Adiprabowo, Willy Fitra Hendria

**Affiliations:** 1 Department of Electronic and Computer Engineering, National Taiwan University of Science and Technology, Taipei 10607, Taiwan; d10602804@mail.ntust.edu.tw; 2 Department of Intelligent Mechatronics Engineering, Sejong University, Seoul 05006, Korea; willyfitrahendria@sju.ac.kr

**Keywords:** FMCW, vital sign, XGBoost, MFCC, COVID-19

## Abstract

During the pandemic of coronavirus disease-2019 (COVID-19), medical practitioners need non-contact devices to reduce the risk of spreading the virus. People with COVID-19 usually experience fever and have difficulty breathing. Unsupervised care to patients with respiratory problems will be the main reason for the rising death rate. Periodic linearly increasing frequency chirp, known as frequency-modulated continuous wave (FMCW), is one of the radar technologies with a low-power operation and high-resolution detection which can detect any tiny movement. In this study, we use FMCW to develop a non-contact medical device that monitors and classifies the breathing pattern in real time. Patients with a breathing disorder have an unusual breathing characteristic that cannot be represented using the breathing rate. Thus, we created an Xtreme Gradient Boosting (XGBoost) classification model and adopted Mel-frequency cepstral coefficient (MFCC) feature extraction to classify the breathing pattern behavior. XGBoost is an ensemble machine-learning technique with a fast execution time and good scalability for predictions. In this study, MFCC feature extraction assists machine learning in extracting the features of the breathing signal. Based on the results, the system obtained an acceptable accuracy. Thus, our proposed system could potentially be used to detect and monitor the presence of respiratory problems in patients with COVID-19, asthma, etc.

## 1. Introduction

On 30 January 2020, the World Health Organization (WHO) officially confirmed that the spread of COVID-19 had caused a global pandemic for countries around the world [1,2]. This pandemic was caused by the SARS-CoV-2 virus [3], which is highly contagious and causes rapid spread through droplets [4,5]. The droplets can spread through the eyes, mouth, or nose within a radius of one or two meters from a person with COVID-19 [6]. The biggest challenge for this pandemic is to control the spread of the virus, and the best strategy to reduce the virus is by preventing direct contact and ensuring social distancing [7,8].

People with COVID-19 usually experience fever and have difficulty in breathing that causes coughing with rapid and short breath (tachypnoea) [9,10,11,12,13]. Therefore, one of the critical conditions that needs to be monitored is the respiration pattern [14,15,16,17,18]. Since pandemic issues, hospitals are always busy and full of patients. Limited medical personnel cause unsupervised care in a hospital [18], whereas some patients suffering from a respiration problem need special or supervised care. Hence, a non-contact respiration monitoring device that can be accessed from a central room in real time is necessary. Thus, radar technology, which provides non-contact detection, has a great opportunity to be developed in the medical field.

Radar sensor has attractive advantages over camera-based systems in terms of light and privacy [18,19,20,21,22,23,24,25,26,27]. Periodic linearly increasing frequency chirp, known as FMCW, is one of the radar technologies that uses a wide frequency bandwidth without requiring wideband processing. FMCW has a simple transceiver architecture, low sampling-rate requirements, low power operation, easier proximity detection, high resolution, and the ability to detect small movements [19,20,28,29,30,31]. Therefore, FMCW radar is capable of detecting the vibration of chest displacement [19,20], which is the result of the lungs’ and heart′s mechanical activity [22].

Several studies have been conducted to obtain an accurate respiration rate [32,33] from chest displacement information. However, patients with a respiration disorder or COVID-19 have an unusual respiration characteristic pattern [13] that cannot be represented by using the respiration rate. Therefore, machine-learning assistance in classifying the breathing pattern plays an important role in detecting respiratory disorder. The addition of machine learning will significantly contribute to the automation of a more sophisticated and more intelligent system. Thus, we tried to incorporate radar technology with machine learning to build a system that can detect and classify the breathing pattern disorder.

Based on the background mentioned earlier, we propose a non-contact breathing pattern detection using FMCW radar with XGBoost classifier and MFCC feature extraction in an indoor environment. Some signal processing steps are implemented to extract the breathing information from chest displacement information. XGBoost classifier and MFCC feature extraction are used to classify the breathing class. XGBoost is often used in machine-learning problems because it combines boosting and gradient boosting so that it can process data quickly [34,35]. Moreover, MFCC feature extraction [36,37] helps the XGBoost to identify, minimize and capture important parts of the signal. 

The proposed system will not be a perfect substitute for a professional doctor. However, it is hoped that our research can help to screen and monitor patients infected by COVID-19.

The classification model was evaluated and obtained a reasonable accuracy—87.375%. The implementation of the proposed system was tested for a real-time operation and successfully detected five different classes of breathing waveform.

The rest of the chapter is summarized as follows: Section 2 describes the related work, Section 3 explains the proposed method, Section 4 demonstrates the experimental result, and Section 5 concludes the work.

## 2. Related Work

The listening technique to listen to the breath sounds using a stethoscope is known as the auscultation technique. The auscultation technique is the basic technique used by doctors to evaluate breath sounds. This technique is quite simple and inexpensive but has a weakness; the analysis results are subjective [38]. Due to these factors, misdiagnosis may occur if the auscultation procedure is not performed properly.

Several studies have been conducted to detect and monitor human body conditions without physical touch, such as using CT scan, X-ray, camera, thermal camera, photoplethysmography technology [39], ultrasound technology, Wi-Fi [40,41,42], radar [43,44,45,46,47,48,49], thermography, etc. [50,51]. 

CT scan [52] and X-ray technology [53] have a high image precision and resolution, but it is quite expensive. CT scanners and X-ray machines are quite large and not portable. It takes a professional to analyze the images. Furthermore, the negative impact is that the patients are exposed to radiation, which is bad for their bodies.

Depth camera technology can be used to observe the chest displacements by recording video footage of the chest movements [13,14,54,55,56]. However, the camera has limitations in terms of light and privacy [18,21,22,23,24,25,26].

In thermography, infrared radiation is commonly used to measure the human body temperature [57]. An infection will usually cause the body temperature to be abnormal. [58]. Additionally, in general, COVID-19 patients have a body temperature above 37 degrees Celsius [59,60].

In [39], non-contact photoplethysmography technology is used to monitor oxygen saturation in the blood (SpO_2_). In estimating SpO_2_, real-time face video monitoring of the patient is carried out with a camera. An abnormal SpO_2_ value is a sign of potential COVID-19 infection.

Another study developed ultrasonic waves for monitoring the movements of organs [61]. The disadvantage of this technology is that patients are not allowed to eat for several hours before the monitoring process is carried out [62].

The breathing rate measurement using Wi-Fi was successfully conducted by using peak detection and with CSI amplitude [41], CSI phase [42], and RSS [40]. Unfortunately, RSS and the amplitude of CSI are not sensitive to the chest motion [40,41]. Furthermore, the measurement accuracy decreases dramatically if the patient location is outside of the specified distance [40,42].

Radar sensor has attractive advantages in monitoring the breathing pattern [63] over camera-based systems in terms of light and privacy [18,21,22,23,24,25,26]. In [63], non-contact vital-sign detection using radar has been developed, and Lee et al. [64] used radar to observe the different breathing patterns. They [63,64] used Doppler radar to capture various breathing patterns, but did not classify them. Ultra-wideband radar (UWB) [65,66,67,68], continuous wave (CW) [21,68,69,70,71], and FMCW are the radar technologies that can be used to develop non-contact medical devices [72,73]. UWB radar has a high resolution and low level of radiation [74]. However, high power is required to transmit the signal during a short pulse period. Meanwhile, CW is unable to detect vibration, making it difficult to detect a small movement. In [21], Doppler radar-based continuous-wave (CW) was used for the automatic breathing pattern classification system using the SVM classifier. The CW radar can measure the relative velocity accurately at a very low transmit power and tiny equipment size. When operating at low transmit power, the range is limited. CW has a weakness in measuring tiny position changes because the signal is not modulated. Besides, other moving objects in front of and behind the target will interfere with the CW signal, making it difficult to distinguish the target from the disturbing object [75].

As mentioned earlier, FMCW has a low-power operation and easier proximity detection [30]. It has a high-resolution speed and the ability to detect tiny movements [76]. One of the advantages of using FMCW is that it has the ability to filter interrupting objects in the range domain. All targets ahead of and behind the selected range can be eliminated through the monitoring process in the frequency domain. The FMCW radar can measure small movements as the signal is modulated. The respiration rate detector with FMCW performs the measurement based on the variation of the phases due to the chest displacement [47,48,49]. Initially, frequency analysis was applied to estimate the distance between the subject and the FMCW radar. Furthermore, feature detection and frequency analysis of phase variance at estimated distances are implemented. In a frequency analysis-based method, the breathing rate is estimated by detecting the peaks due to respiration over a spectrum [46]. The studies on estimating the breathing rate using radar have been investigated extensively with Doppler radar [21,43,45,46] and FMCW radar [47,48,49]. Previous studies on Doppler and FMCW radar provide an accurate estimation of respiration rate [43,44,45,46,47,48,49].

The current state-of-art literature shows that CT scan and X-ray have a good precision but are expensive and cannot be used in real time; cameras, thermal cameras, and photoplethysmography can be used in real time but are not good in terms of privacy and require good lighting; ultrasound technology and Wi-Fi technology are less sensitive and not easy to use; UWB and CW radars are sensitive but require a lot of power. Thus, the aforementioned solutions are less applicable for real-time monitoring of the condition of COVID-19 patients in quarantines or hospitals. 

On the other hand, FMCW radar technology allows real-time and non-contact measurement, maintains privacy, is not affected by light, has a simple transceiver architecture, has a wide frequency with low power consumption, has a low sampling-rate requirement, has easier proximity detection, can filter interrupt objects, and has a high resolution, which is very important for detecting vibration. 

For this reason, the most suitable sensor to overcome all of the aforementioned problems is to use FMCW radar technology. FMCW is a good choice for implementing non-contact respiration detection for COVID-19 patients. 

## 3. Proposed System

**Figure 1:**
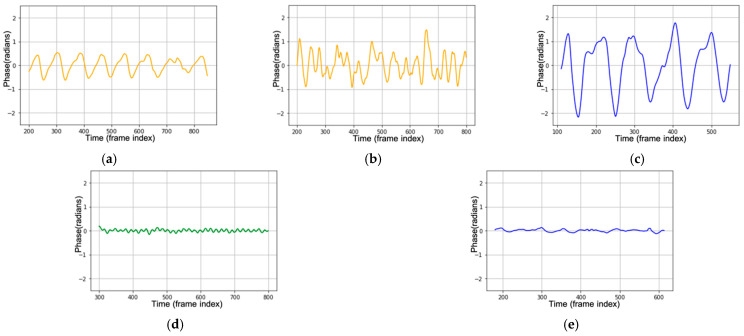
Breathing waveform in the time domain for (**a**) normal breathing; (**b**) deep and quick breathing; (**c**) deep breathing; (**d**) quick breathing; (**e**) holding the breath, recorded by TI-IWR 1443.

This section explains how the non-contact monitoring and classification of breathing patterns using the XGBoost classifier and MFCC feature extraction using FMCW works. Before we begin, we start by formally defining five classes of breathing patterns as follows:

- Class 1—normal breathing: normal breathing has a constant breathing waveform and similar pattern during the time, shown in Figure 1a.
- Class 2—deep and quick breathing: deep and quick breathing has a large amplitude with a high frequency (high respiration rate), shown in Figure 1d.
- Class 3—deep breathing: deep breathing has a large amplitude with a normal respiration rate, shown in Figure 1c.
- Class 4—quick breathing: quick breathing has a small amplitude (short breath) with high frequency (high respiration rate), shown in Figure 1d.
- Class 5—holding the breath: the breathing waveform is almost disappeared, and the amplitudes are close to zero, shown in Figure 1e.

Class 1 shows us the normal breathing of an adult. In general, 12 to 20 breaths per minute is the average respiration rate for a relaxed adult. For class 2 to 5, we chose those four breathing patterns because each class has similarities with the symptoms of several diseases.

Breathing pattern disorders are abnormal breathing patterns associated with excessive breathing. They range from simple upper-chest breathing to the most extreme scale, hyperventilation (HVS) [77]. Usually, hyperventilation sufferers experience deep and rapid breathing such as class 2, deep and quick breathing. In general, sufferers of this respiration pattern disorder experience chronic or recurring changes in their breathing patterns that cannot be attributed to a specific medical diagnosis. When ventilation exceeds metabolic requirements, it results in chemical and hemodynamic changes that lead to a breathing pattern disorder. Class 2 (deep and quick breathing) can be found in Kussmaul and Biot patients. The Kussmaul and Biot breathing occur in patients who experience deep and rapid breathing. This indicates that the organs are becoming too acidic. It is caused by kidney failure, metabolic acidosis, and diabetic ketoacidosis. The body breathes quickly and deeply to release carbon dioxide, which is an acidic compound in the blood [78].

In the medical field, class 3 (deep breathing) is known as hyperpnea. Hyperpnea is an increasing depth of breath at normal frequencies.

Asthma starts with a cough or a wheeze. Usually, the chest feels tight, the breathing speeds up, and it becomes shallower. It will cause the person to feel short of breath. These are common symptoms of an asthma attack, which is related to class 4—quick breathing. COVID-19 and tachypnoea patients sometimes have unexpectedly short breathing at unexpected times related to class 4, quick breathing, or short breathing. This kind of patient needs supervised care because short breathing may occur suddenly. This critical condition is very risky for their life.

Bradypnea is a decreased frequency of breath or slowed breathing related to class 5—holding the breath. This situation is found in respiratory center depression. Bradypnea is usually found in patients who use alcohol or narcotics and in patients with tumors. Besides, patients who have difficulties in breathing and are about to die also have a breathing waveform such as class 5.

**Figure 2:**
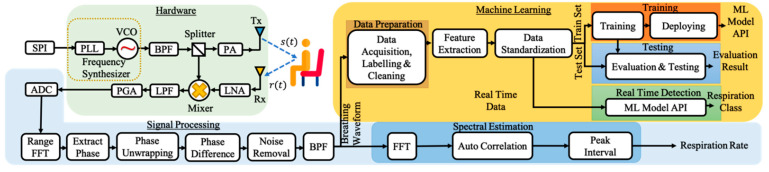
Block diagram of the proposed system.

Now, we will explain how our proposed system works. Figure 2 illustrates the system model that detects and classifies the breathing pattern based on FMCW radar. In general, we have three modules. The first module is the FMCW module that generates and receives the FMCW signal. The first module is explained in the first sub-section. The second module, which is presented in the second sub-section, is the signal processing module that processes and extracts the signal into a breathing waveform. The third sub-section explains the last module, the machine learning module. The machine learning module trains and tests the data and generates the machine-learning model for classification.

### 3.1. FMCW Module

#### 3.1.1. Signal Processing in Hardware

In this part, we explain the signal processing step for generating the FMCW signal and obtaining the reflected signal. The steps are described in the following sub-paragraph.

- The process begins when the user instructs the microcontroller unit (MCU).
- The instruction is transmitted through a serial peripheral interface (SPI), serial communication for short-distance communication.
- FMCW uses a continuous signal that has modulated frequency. Thus, we need a frequency synthesizer that generates the modulated frequency signal.
- A phase-locked loop (PLL) is a feedback control system that compares the phase of two input signals in a frequency synthesizer. It produces an error signal proportional to the difference between their steps.
- The error signal is then passed through the low-pass filter (LPF) and is used to drive the voltage control oscillator (VCO).
- The VCO produces the output frequency. VCO increases the frequency by increasing the voltage.
- Bandpass filter (BPF) is then used to filter the signal. The signal is passed through a BPF so that only the main frequency is used and the harmonic frequency is ignored.
- The splitter is used to split the signal for the mixer and the transmitter.
- A power amplifier (PA) amplifies the signal before being transmitted by the transmitter antenna (Tx).
- Tx emits a modulated signal s(t) towards the object. The object will reflect the signal, and the receiver will receive the reflection.
- The signal r(t) received by the receiver will have a difference in frequency compared to the signal emitted by the transmitter. This difference describes the time for the signal to travel from the transmitter to the object. The object distance is obtained from the traveling time.
- As the received signal is very weak, we use a low noise amplifier (LNA) that amplifies the received signal r(t).
- The mixer will mix the transmitted signal s(t) and received signal r(t).
- We only need the signal with low frequency; we pass the signal through LPF to obtain the low-frequency signal and remove the high-frequency signal.
- PGA is a programmable gain amplifier that can control the gain.
- Finally, the data is transmitted to the MCU.
- The analog-to-digital converter (ADC) will convert the analog signal to the digital signal.

This study uses a TI-IWR 1443 mm-Wave sensor from Texas Instruments [79] to measure the chest displacements. This study was carried out using FMCW radar with a starting frequency of 77 GHz and a chirp frequency of 4 GHz. 

As mentioned in the previous section, FMCW has the ability to detect the presence of very small displacements. Usually, the chest displacement has an amplitude below 10 mm with a low frequency of less than 4 Hz. Therefore, there is no large phase change during the time (fast time). Phase changes can be seen from successive chirps (slow time). In Equation (12) of paper [44], if an object is at a distance R, then:

(1) $$\phi_{b}=\phi_{c}+\frac{4\pi R}{\lambda },$$

where $\phi_{b}$ is the phase shift at the receiver; $\phi_{c}\;$is the phase, which is constant for a fixed object; and λ is the wavelength. From Equation (1), it is shown that a smaller λ will result in a larger phase change. This explains why 77 GHz, the smaller wavelength millimeter-wave radar (≈3.9 mm), can measure ten-micron vibrations caused by the lungs and heart. For an object with static angles placed at a fixed distance, they [44] experimentally determined the phase sensitivity by measuring the phase variation across the object-bin range as a function of time. Their study showed that at SNR >40 dB, phase sensitivity <7 milli-radians corresponds to a displacement sensitivity of ≈2 microns. Thus, we know that 77 GHz wave radar has greater sensitivity in measuring small displacements. This gives us confidence that using the same device, the system is capable of measuring 10-micron vibrations for breathing measurements. In order to detect the small scale of displacement, the sensor measures the change in phase of the FMCW signal. The sensor detects the chest displacement when it is located nearby the person sitting around the sensor.

#### 3.1.2. FMCW Signal Model

**Figure 3:**
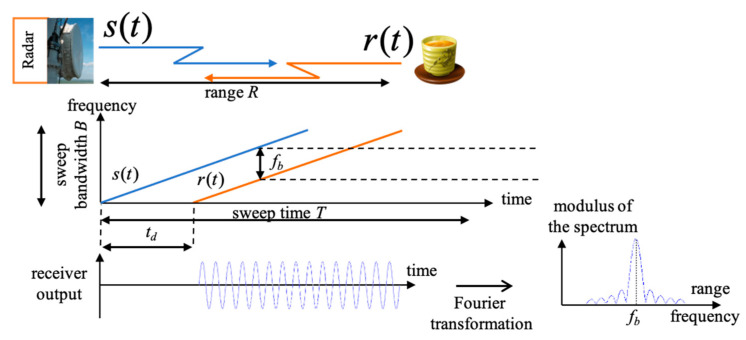
FMCW basic concept.

In theory, the FMCW signal model has been explored in several previous studies [80]. This part will briefly explain the basic FMCW signal model that we use in the system. FMCW signal transmits a signal with periodic frequency modulation. The frequency increases linearly over the length of the sweep time T, as shown in Figure 3.

Based on similar triangles in Figure 3, we have the received time $t_{d}=\frac{2R}{c},$ so $\frac{t_{d}}{T}=\frac{f_{b}}{B}$, where R is the distance, $f_{b}$ is the beat frequency, c is the light speed and B is the sweep bandwidth. The distance $R=\frac{cTf_{b}}{2B}$ can be obtained from $f_{b}=\frac{1}{T}$. The distance resolution is $dR=\frac{c}{2B}$. Note that frequency $\frac{1}{2\pi }\frac{d}{dt}\;\left(2\pi f_{c}t+\pi \frac{B}{T}t^{2}\;\right)=f_{c}+\frac{B}{T}t\;$. The transmitted FMCW signal is expressed as follows:

(2) $$s\left(t\right)=A_{T}\cos\left(2\pi f_{c}t+2\pi \frac{B}{T}\int_{-\infty }^{t}\tau \;d\tau +\phi \left(t\right)\right)=A_{T}\cos\left(2\pi f_{c}t+\pi \frac{B}{T}t^{2}+\phi \left(t\right)\right),$$

where $\;A_{T}$ is the transmitted signal power, $f_{c}$ is the starting frequency of the chirp, and ϕ(t) is the phase. The received signal is the delay time of the transmitted signal defined as:

(3) $$r\left(t\right)=\alpha A_{T}\cos\left(2\pi f_{c}\left(t-t_{d}\right)+\pi \frac{B}{T}{\left(t-t_{d}\right)}^{2}+\phi \left(t-t_{d}\right)\right),$$

with α as the resized scale. The mixer output is:

(4) $$s\left(t\right)r\left(t\right)=\alpha {\left(A_{T}\right)}^{2}\cos\left(2\pi f_{c}t+\pi \frac{B}{T}t^{2}+\phi \left(t\right)\right)\cos\left(2\pi f_{c}\left(t-t_{d}\right)+\pi \frac{B}{T}{\left(t-t_{d}\right)}^{2}+\phi \left(t-t_{d}\right)\right)\;\begin{array}{c} =\frac{\alpha {\left(A_{T}\right)}^{2}}{2}\left(\cos\left(4\pi f_{c}t-2\pi \frac{Bt_{d}}{T}t+2\pi \frac{B}{T}t^{2}+\pi \frac{B}{T}{\left(t_{d}\right)}^{2}-2\pi f_{c}t_{d}+\phi (t)+\phi \left(t-t_{d}\right)\right)\right.\\ \left.+\cos\left(2\pi \frac{Bt_{d}}{T}t+2\pi f_{c}t_{d}-\pi \frac{B}{T}{\left(t_{d}\right)}^{2}+\phi (t)-\phi \left(t-t_{td}\right)\right)\right) \end{array}$$

The LPF output is:

(5) $$b\left(t\right)=LPF\left(s\left(t\right)r\left(t\right)\right)=\frac{\alpha {\left(A_{T}\right)}^{2}}{2}\cos\left(2\pi \frac{Bt_{d}}{T}t+2\pi f_{c}t_{d}-\pi \frac{B}{T}{\left(t_{d}\right)}^{2}+\Delta \phi \right),$$

where Δϕ is the residual phase noise. Suppose that the target is stationary, let $t_{d}=\frac{2R}{c},\;f_{c}=\frac{c}{\lambda }$ into b(t)

(6) $$\begin{array}{c} =\frac{\alpha {\left(A_{T}\right)}^{2}}{2}\cos\left(2\pi \frac{B}{T}\frac{2R}{c}t+2\pi f_{c}\frac{2R}{c}-\pi \frac{B}{T}{\left(\frac{2R}{c}\right)}^{2}+\Delta \phi \right)\approx \frac{\alpha {\left(A_{T}\right)}^{2}}{2}\cos\left(2\pi \frac{B}{T}\frac{2R}{c}t+\frac{4\pi f_{c}R}{c}+\Delta \phi \right)\\ =\frac{\alpha {\left(A_{T}\right)}^{2}}{2}\cos\left(2\pi \frac{B}{T}\frac{2R}{c}t+\frac{4\pi R}{\lambda }+\Delta \phi \right)=\frac{\alpha {\left(A_{T}\right)}^{2}}{2}\cos\left(2\pi f_{b}t+\Delta \phi \right). \end{array}$$

Note that frequency: $f_{b}=\frac{1}{2\pi }\frac{d}{dt}\left(2\pi \frac{B}{T}\frac{2R}{c}t+\frac{4\pi R}{\lambda }\;\right)=\frac{B}{T}\frac{2R}{c}.\;$The beat signal means the receiving signal, which is the result of mixer and LPF filter. Thus, we have:

(7) $$b\left(t\right)\approx \frac{\alpha {\left(A_{T}\right)}^{2}}{2}\cos\left(2\pi \frac{B}{T}\frac{2R}{c}t+\frac{4\pi R}{\lambda }+\Delta \phi \right)=\frac{\alpha {\left(A_{T}\right)}^{2}}{2}\cos\left(2\pi f_{b}t+\phi_{b}+\Delta \phi \right),$$

where $\phi_{b}$ is the phase of the beat signal. The beat signal b(t) contains information about the frequency difference, determining the distance R between the radar and the target. The maximum detection range of FMCW is $R_{max}=\frac{cTf_{b}}{2B}\;$and the minimum detection range of FMCW is $R_{min}=\frac{c}{2B}$.

### 3.2. Signal Processing Module

#### 3.2.1. Range FFT

After passing through the low-pass filter mentioned above, the beat signal is sampled in the fast time-frequency. Then, the fast Fourier transformation (FFT) range is implemented to obtain the spectrum. The peak value of the signal spectrum defines the target distance. Peak detection is performed to determine the difference in frequency and distance between the radar and the target. Chest movements caused by heart and lung activity can be observed when the body is in a constant state. The phase change $\Delta \phi_{b}$ of the beat signal can represent the small-scale vibration ΔR because it has a positive linear relationship.

FFT ranges are referred to as complex span profiles. These FFT ranges are aggregated in a slow-span time matrix for each time T. The variation in the distance from the radar to the chest surface is proportional to the change in phase received by the receiver. The slow time span matrix is then sent to the processor on the PC, and signal extraction is performed, as shown in Figure 2.

The chest surface displacement due to vital organ vibrations has a small amplitude ranging from <12 mm with a low frequency of <4 Hz. This indicates no drastic change in phase during the span of the chirp time (fast time axis) so that the chest movement can be observed by measuring the phase change between successive chirps (slow time axis).

This paragraph describes the slow-time axis sampling rate considerations. Following the Nyquist criteria [44] of the theory, the sampling rate must be twice the sampling rate of maximum frequency to prevent noise aliasing. As the observed range of vibrations is between 0.1 and 4 Hz, the used sampling rate is 20 Hz. On the other hand, the sampling rate must be large to cover up the phase redundancy. In theory, for an object vibrating with $A\;sin\;\left(2\pi f_{m}t\right)$, the selected slow-axis sample must satisfy $Fs>\frac{8\pi f_{m}A}{\lambda }$. A is the amplitude and $f_{m}$ is the vibration frequency. For the chirp duration, we chose 50 μs for one chirp range. Based on the theory, SNR and displacement sensitivity are better achieved when the chirp duration is higher.

#### 3.2.2. Extraction and Unwrapping

To obtain information on the value of the displacement distance, arctangent and unwrapping operation on the phase value are calculated as $\varphi \;\left(m\right)=unwrap\;\left[\tan^{-1}\frac{Q}{I}\right]$. I and Q are measured signals of I channel and Q channel, respectively.

The obtained phase is in radian. This phase information can be any real value wrapped into the interval 2π with domain ]−π, π] by the arctan operator. This information is limited between −π and π. This condition causes a phase ambiguity for calculating the phase cycle. To solve this problem, an unwrapping process, a process to eliminate the phase ambiguity, should be carried out so that an absolute phase is obtained. Phase unwrapping reconstructs a continuous signal by removing some 2π ambiguity.

To measure tiny vibrations, the change of the signal within time is measured. From Equation (7), if an object changes position along ΔR, then the phase change between successive measurements is given by:

(8) $$\Delta \phi_{b}=\frac{4\pi \Delta R}{\lambda }$$

where $\Delta \phi_{b}$ is the phase change of the beat signal, ΔR is the change of the distance, and λ. is the wavelength. The phase can be measured by taking the FFT of signal b(t) and calculating the phase over the object range. The distance can be calculated by the equation R=λ (ϕ+k) where k is the phase ambiguity that must be obtained through the phase unwrapping process in order to obtain the absolute phase.

“Itoh’s condition” theory [81], adopted by most phase unwrapped strategies [82], is that the absolute value of the phase difference between adjacent neighbors in a continuous phase signal is less than π for unambiguous phase wrapping. When Itoh’s condition is not violated, it is possible to obtain absolute and constant values easily. Let us define the wrapper operator W (·) that wraps any phase ϕ. into ]−π, π] by

(9) W: ℝ⟼ ]π, π]ϕ↪ϕ−2πk,

where k∈ℤ, such that it follows the following rule:

(10) $$\phi \left(t-1,t\right)=\{\begin{array}{c} \Delta \phi_{t},\;\;\;\;\;\;\;\;\;\;\;\;\;\;\;\;\;\;if\;\left|\Delta \phi_{t}\right|<\pi \\ \Delta \phi_{t}+2\pi \;\;\;\;\;\;\;\;if\;\Delta \phi_{t}\leq -\pi \\ \Delta \phi_{t}-2\pi \;\;\;\;\;\;\;if\;\Delta \phi_{t}\geq -\pi \end{array}$$

(11) $$\Delta \phi_{t}=\phi \left(t\right)-\phi \left(t-1\right),$$

where ϕ(t) is the current phase and ϕ(t−1) is the previous phase. Thus, Itoh’s condition [81] can be represented by:

(12) $$\left|\Delta \phi_{t}\right|\leq \pi .$$

Then, we have:

(13) $$\overset{m}{\underset{t=1}{\sum }}\Delta \phi_{t}=\phi \left(m\right)-\phi \left(0\right).$$

From Equation (9), we have $W\left(\phi \left(t\right)\right)=\phi \left(t\right)-2\pi k_{t},\;$with $k_{t}\in \mathbb{Z}$, so:

(14) $$\Delta W\left(\phi \left(t\right)\right)=\phi \left(t\right)-\phi \left(t-1\right)-2\pi (k_{t}-k_{t-1}),$$

where $k_{t}-k_{t-1}\in \mathbb{Z}$. Then, we can write Equation (11) as:

(15) $$\underset{p}{\underbrace{W\left[\Delta W\left(\phi \left(t\right)\right)\right]}}=\Delta \phi_{t}\underset{q}{\underbrace{-2\pi (k_{t}-k_{t-1})-2\pi k}},$$

where $k_{t},k_{t-1},k\in \mathbb{Z}$, and 2πk is the proper 2π multiple to bring p into the principal interval. From Equation (12) and |p|≤π, we have q=0, so that we can write:

(16) $$W\left[\Delta W\left(\phi \left(t\right)\right)\right]=\Delta \phi_{t}$$

Finally, from Equations (13) and (16), we obtain:

(17) $$\phi \left(m\right)=\overset{m}{\underset{t=1}{\sum }}W\left[\Delta W\left(\phi \left(t\right)\right)\right]+\phi \left(0\right).$$

From Equation (17), we can obtain the unwrapped phase at any time t from the wrapped phase value, with its absolute phase value ϕ(0). Thus, we can calculate the absolute phase value for each time when Itoh′s condition is met. Lastly, the phase difference between successive unwrapped phases is calculated.

#### 3.2.3. Noise Removal

Noise-induced phase wrapping error might corrupt the un-wrapped differential phase a (m), especially in phases around −π or π. By calculating the phase difference backwards a (m)−a (m−1) and forwards a (m)−a (m+1), impulse-like noise can be eliminated. If the phase exceeds a certain limit, the a (m) value is replaced with an interpolation value.

#### 3.2.4. IIR BPF Using Cascaded Bi-Quad

The chest displacement due to cardiac and breathing activity is represented by two overlapping sinusoidal signals, where one represents the heart waveform and the other represents the respiratory waveform. Generally, the adult chest moves due to the process of respiration activity with an amplitude of 4 to 12 mm at a frequency between 0.1 and 0.5 Hz, and cardiac activity with a frequency between 0.8 and 4 Hz with an amplitude of 0.2 to 0.5 mm [83]. Chest surface fluctuations caused by pulmonary and cardiac motion are modeled as a signal [22], as follows:

(18) $$x\left(t\right)=\overset{J}{\underset{i=1}{\sum }}a_{ri}\;\cos\left(2\pi if_{r}t+\theta_{ri}\right)+\overset{K}{\underset{i=1}{\sum }}a_{hi}\cos\left(2\pi if_{h}t+\theta_{hi}\right).$$

The amplitude of respiration and heart waveform for the i-th harmonic component is denoted as $a_{ri}$ and $a_{hi}$, respectively. $f_{r}$ is the base frequency of the respiratory waveform and $f_{h}$ is the base frequency of the heart waveform. The harmonic phase sequences of the respiratory and heart signal are $\theta_{ri}$ and $\theta_{hi}$, respectively. Finally, J and K are the total numbers of components.

As mentioned earlier, the respiration and heart waveform have different frequency bands so that suitable frequency filters can separate them. In this study, a fourth-order IIR cascade Bi-quad BPF was used to obtain a respiratory signal in the frequency range between 0.1 and 0.5 Hz. 

The BPF is a frequency filter that passes signals within a certain frequency range. The signal is passed between the lower limit frequency to the upper limit frequency. In other words, this BPF will reject or attenuate frequency signals that are outside the specified range.

Increasing the Butterworth filter order allows for faster roll-off around the cutoff frequency while maintaining flatness in the stopband and passband. However, direct application of a high-order recursive filter will cause different coefficients in many order quantities. Besides, this makes the practical application difficult [84]. Thus, a cascaded bi-quad is used to avoid the use of a high-order filter.

In this section, we explain how the Bi-quad BPF works. The pole-zero form of the BPF response [85] is described as follows:

(19) $$H\left(z\right)=K\frac{{\left(z+1\right)}^{N}{\left(z-1\right)}^{N}}{\left(z-p_{1}\right)\left(z-p_{2}\right)\ldots \left(z-p_{2N}\right)}.$$

N is the order of the BPF. Next, H(z) is converted into cascaded sections (bi-quads). Thus, H(z) can be written as the product of N sections with complex-conjugate poles as follows:

(20) $$H\left(z\right)=K_{1}\frac{\left(z+1\right)\left(z-1\right)}{\left(z-p_{1}\right)\left(z-p_{1}^{*}\right)}.K_{2}\frac{\left(z+1\right)\left(z-1\right)}{\left(z-p_{2}\right)\left(z-p_{2}^{*}\right)}.\;\;\;\ldots \;\;\;.\;K_{N}\frac{\left(z+1\right)\left(z-1\right)}{\left(z-p_{N}\right)\left(z-p_{N}^{*}\right)}\;.$$

$p_{k}^{*}$ is the complex conjugate of $p_{k}$. At each bi-quad, a zero is assigned at z=+1 and z=−1. We label each term in the equation as biquadratic because it has a quadratic numerator and denominator. Furthermore, we can extend the numerator and denominator of the k-th bi-quad section as follows:

(21) $$H_{k}\left(z\right)=K_{k}\frac{z^{2}-1}{z^{2}-\left(p_{k}+p_{k}^{*}\right)z+p_{k}p_{k}^{*}\;}=K_{k}\frac{z^{2}-1}{z^{2}+a_{1}z+a_{2}}.$$

$a_{1}=-2*real\left(pk\right)\;$and $a_{2}={\left|pk\right|}^{2}$. After dividing the numerator and denominator by $z^{2}$, we form the following equation:

(22) $$H_{k}\left(z\right)=K_{k}\frac{1-z^{-2}}{1+a_{1}z^{-1}+a_{2}z^{-2}}.$$

Since the same zero is assigned for each bi-quad, the feed-forward (numerator coefficient) b = [1 0 −1] will be the same for all N bi-quads. So, we get:

(23) $$a=\left[1\;-2*real\left(pk\right)\;{\left|pk\right|}^{2}\right],\;b=\left[1\;0\;-1\right].$$

A pair of complex conjugate poles is not sufficient to define a second-order polynomial. For a BPF, after bilinear transformation, the output has to be scaled to achieve unity gain in the passband [85]. Each bi-quad is allowed to have a gain of 1 at the filter geometric mean frequency $f_{0}$. for finding the gain $K_{k}$. Then, $H_{k}\left(z\right)$ is evaluated at $f_{0}\;$and $K_{k}$ [85], set as follows:

(24) $$K_{k}=\frac{1}{\left|H\left(f_{0}\right)\right|}.$$

To find $f_{0}$, we define $f_{1}=f_{center}\;–\frac{bw}{2}$ and $f_{2}=f_{center}+\frac{bw}{2}$. Thus, $f_{0}=\sqrt{f_{1}*f_{2}}$. For a narrowband filter, $f_{0}$ is close to $f_{center}$. In theory, we can arrange the sequence of the bi-quad freely. However, to reduce and minimize the possibility of clipping, a bi-quad with the peaking response should be put at the end.

**Figure 4:**
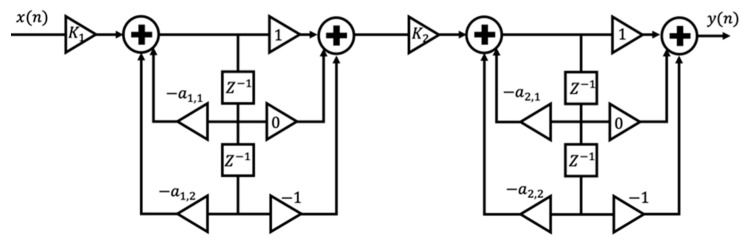
Fourth order of IIR BPF using cascaded bi-quad.

As our system uses a fourth-order IIR cascaded Bi-quad BPF, we need to cascade two IIR bi-quad BPF, as shown in Figure 4. Based on Equations (23) and (24), we have the denominator coefficient a, nominator coefficient b and the gain $K_{k}$, respectively, as follows:

(25) $$a=\left[\begin{array}{ccc} 1 & a_{1,1} & a_{1,2}\\ 1 & a_{2,1} & a_{2,2} \end{array}\right]=\left[\begin{array}{ccc} 1 & -1.963 & 0.964\\ 1 & -1.85 & 0.868 \end{array}\right],\;b=\left[b_{0}\;\;b_{1}\;\;b_{2}\right]=\left[1\;\;0\;-1\right]$$

(26) $$K=\left[\begin{array}{cc} 0.116 & 0.031 \end{array}\right]$$

**Figure 5:**
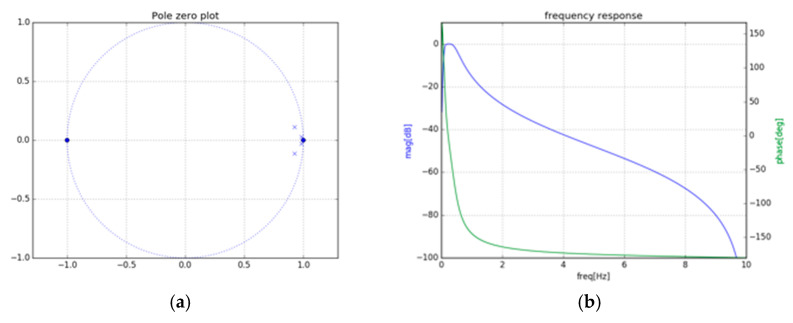
(**a**) Pole-zero plot, and (**b**) frequency response for fourth-order IIR cascaded Bi-quad BPF.

Figure 5a presents the pole-zero plot, and Figure 5b illustrates the frequency response of the fourth-order IIR cascaded Bi-quad BPF for frequency 0.1 to 0.5 Hz.

**Figure 6:**
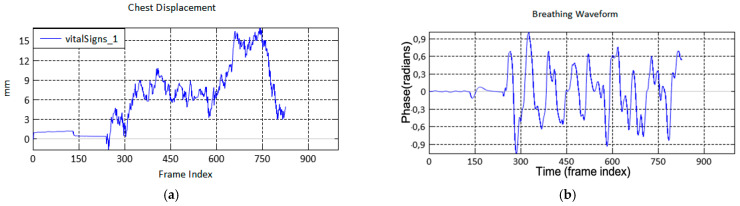
(**a**) Unwrapped phase after the phase difference and noise removal, labelled as the chest displacement; (**b**) the output of IIR BPF is the breathing waveform.

One of the measurement samples shows the unwrapped phase after the phase differences operation, and noise removal is represented in Figure 6a as the chest displacement. Then, the signal is passed through the fourth-order of IIR BPF using a cascaded bi-quad. Note that the breathing waveform becomes more obvious, as shown in Figure 6b.

#### 3.2.5. Respiration Rate

In order to verify that the breathing waveform is correct, we also calculate the breathing rate. The breathing waveform was passed through a spectrum estimation, autocorrelation, and interpeak distance block to estimate the breathing rate. The BPF is employed to eliminate the noise. Peak detection is performed to determine the difference in frequency and the distance between the radar and the target. The respiration rate value is obtained by calculating the distance between the respiratory wave signal peaks in the time domain.

### 3.3. Machine Learning (Classification Method) Module

The proposed method uses the XGBoost model as the classifier and MFCC as the feature extraction. We explain the machine-learning module as several parts as follows.

#### 3.3.1. Pre-Processing

When recording the respiratory data, some pieces of data have 0 values or missing values. Besides, some data do not represent the desired class. For example, when the system started to record, the subject had not started imitating the suitable breath class. Thus, data that does not represent the suitable class has 0 value or missing values and is discarded from the data set.

Data sets contain some features that differ in unit and range. Before the data processed by a machine-learning algorithm, data sets must be converted into a proper format. If standardization is not implemented, large numbers and a wide range of features will reach more weight than features with a small number and small range. It means that features with a large number and range will obtain more priority. Therefore, to suppress all these effects, it is necessary to scale the feature with a standardization process. Standardization facilitates faster convergence of loss functions for some algorithms.

(27) $$z=\frac{x-\mu }{\sigma }$$

For each piece of data, we limit each window to 5 s and segment it with 85 step size.

#### 3.3.2. MFCC Feature Extraction

**Figure 7:**
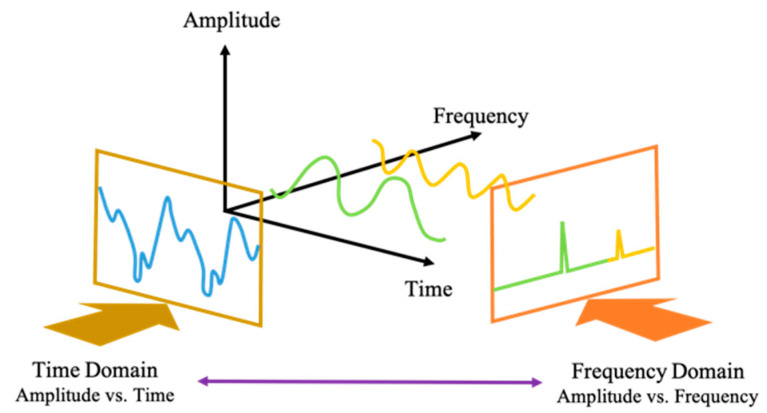
Component of breathing waveform.

The Mel-frequency cepstral coefficient (MFCC) is a feature extraction introduced by Davis and Mermelstein around 1980 [36,37]. In order to improve the classification accuracy, MFCC feature extraction converts signal waves into cepstral coefficients. It converts the signal into several vectors to generate vector features [86]. The MFCC of a signal is a small set of features with a value between 10 and 20, representing a spectral envelope of the overall shape. The advantage of MFCC is that it can minimize and capture the important parts of the signal. MFCC works based on the differences in frequency [87,88]. MFCC is widely used in audio/speech recognition. We adopt MFCC because the breathing waveform is similar to the audio signal, which has a three-dimensional signal in time, amplitude, and frequency, as shown in Figure 7. Most of the audio recognition studies use MFCC because it has the best performance in extracting the signal. The study in [89] shows good training and test results in speech recognition using MFCC [89]. Thus, in our study, we employ MFCC to assist machine learning in extracting the breathing waveform.

**Figure 8:**
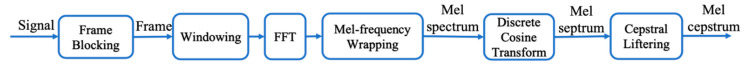
MFCC feature extraction technique.

MFCC stages, shown in Figure 8, start from frame blocking, windowing, FFT, Mel-frequency wrapping (MFW), discrete cosine transform (DCT), and cepstral liftering.

1. Frame Blocking

Frame blocking divides the signal into several frames then makes the frames overlap each other. The signal is divided into U samples and shifted by V samples so that U=2V with V<U. The width of the frames is denoted by U, while the width of each frame is shifted as V. The overlap width is calculated as the difference of U−V.

2. Windowing

Windowing is necessary because the effect of frame blocking on signals causes discontinuity. One way to avoid a discontinuity at the end of the window is to tap the signal to zero or near zero, thereby reducing errors.

3. Fast Fourier Transform (FFT)

After passing through frame blocking and windowing, FFT is applied to the signal. FFT converts the signal from the time domain to the frequency domain as the spectrum.

4. Mel-frequency Wrapping (MFW)

Mel-frequency wrapping is processed based on a filter bank and produces a mel spectrum. A filter bank is a filter to determine the amount of energy from a certain frequency band. The mel frequency scale is a linear frequency scale at frequencies below 1000 Hz and is a logarithmic scale at frequencies above 1000 Hz. This block wraps the resulting spectrum from FFT so that it becomes a mel scale. The inner frequency range is very wide, and the signal does not follow a linear scale, so the computed spectrum of data is mapped in mel scale using overlapping triangular filters. MFW calculation [36] follows:

(28) $$Y\left[i\right]=\overset{G}{\underset{j=1}{\sum }}T\left[j\right]H_{i}\left[j\right].$$

Y[i] is the calculation result of the mel frequency wrapping at i-th, where 1 ≤ i ≤ E; E is the number of filter bank channels. G is the total magnitude spectrum; T [j] is the result of FFT; Hi [j] is the filter bank coefficient at frequency j. In this case, mel uses a frequency with the mel scale [90] that follows:

(29) $$mel\left(f\right)=2595\log_{10}\left(1+\frac{f}{700}\right),$$

with f as the frequency.

5. Discrete Cosine Transform (DCT)

The DCT produces septrum mel. DCT is assumed to replace the inverse Fourier transform in the MFCC feature extraction process. DCT has the aim of creating septrum mel to improve the quality of recognition. DCT [36] uses the following equation:

(30) $$C_{m}=\overset{K}{\underset{k=1}{\sum }}\left(\log_{10}Y\left[k\right]\cos\left(m\left(k-\frac{1}{2}\right)\frac{\pi }{K}\right)\right),$$

where m=1,2,…,K. $C_{m}$ is the coefficient, Y [k] is the output of the filter bank process on the index k, m is the number of coefficients, and K is the number of expected coefficients.

6. Cepstral Liftering

Cepstral liftering is the last MFCC process that converts the frequency domain signal into the time domain. The cepstral coefficient uses the following equation:

(31) $$w\left(b\right)=1+\frac{C}{2}\sin\frac{b\pi }{C},$$

where b=1,2,…,C.w(b) is the window function to the cepstral features, C is the cepstral coefficients, and b is the cepstral coefficients index. The cepstral liftering is obtained in the form of frames and cepstral coefficients.

#### 3.3.3. Classification Using XGBoost Classifier

One technique that can be used to improve the performance and the confidence level of learning outcomes is using more than one learning algorithm. In ensemble learning, similar learning algorithms generate several hypotheses, and the results are combined to make the predictions. This combination method can minimize learning errors caused by noise, bias, and variations. Usually, these errors occur in learning processes that use unstable classifiers, such as decision trees [91]. XGBoost, which stands for extreme gradient boosting, is an ensemble machine learning technique that uses a gradient enhancement framework for machine learning predictions [34]. XGBoost has a fast execution time and good scalability. XGBoost is a special implementation of gradient boosting. It is called gradient boosting because gradient descent is used. It minimizes errors when forming a new model. By adding the boosting method, it is expected that the classifier performance will increase. Improving the boosting technique at the training stage helps to optimize the weight gain process in machine learning [91].

To understand how XGBoost works, first, we need to understand how the adaptive boosting (AdaBoost) and gradient boosting machine (GBM) algorithms work, which are the basis of XGBoost. AdaBoost works by constructing a weak learner model, namely a tree, and giving each observation the same weight [91]. The obtained tree is then evaluated to see its predictive ability. There will be some incorrect observations for the prediction tree. The weight of incorrect observation will be increased in the next iteration. Thus, we hope that it will be able to predict in the next iteration model accurately. The procedure is repeated so that 10 to hundreds of weak learners are obtained. The final model is decided by combining various trees obtained by a certain weighting mechanism. This AdaBoost approach is classified as a sequential learning process because it sequentially changes the weak learner model. It does not process the parallel tree, such as the random forest algorithm [91]. The GBM algorithm also performs the iterative and sequential method as well as AdaBoost. The prediction of one iteration is obtained by combining the models from the previous iterations.

Furthermore, in each iteration, the model attempts to correct the previous error. The residue of the previous prediction model is used as the response variable for the next model. At each iteration, a loss function is minimized according to the needs of the user for obtaining a classification model. For modeling the regression, the loss function can be estimated by calculating the error sum of squares, whereas, in the general classification, the logarithmic loss function is used. The final prediction is determined by combining all model predictions from all iterations. XGBoost is an extension of the GBM algorithm with several additional features that are useful in speeding up the computation process and preventing overfitting. XGboost can optimize memory and cache usage on a computer so that it can work efficiently, even dealing with large data sizes [34,35]. This feature allows XGboost to run faster than other advanced models such as deep learning and random forest. Meanwhile, the prevention of overfitting is carried out by providing a penalty component to the loss function. In this way, the algorithm will avoid too complex models but poorly perform in predicting events with new data. 

In this part, we explain the choice of our machine learning algorithm. In [35], six different classification algorithms were compared for emotion recognition from Electroencephalography (EEG) signals. The EEG signal used was a one-dimensional signal that changed with the time, as well as our breathing waveform. In their paper, they explained that the algorithm they needed was a fast and accurate algorithm for a real-time prediction. From the Naive Bayes, KNN, C4.5, Decision Tree, Random Forest, and XGBoost algorithms, XGboost achieves the best accuracy for classifying four classes compared to five other classification algorithms [35]. Additionally, in [92], the performance between XGBoost and Light GBM was tested, showing that XGboost has shown much better accuracy and outperforms existing boosting algorithms [92]. XGBoost combines several algorithm techniques that can minimize the learning error. As mentioned in the previous paragraph, XGboost uses the concept of AdaBoost and GBM. It does not process parallel trees such as random forest [91,93]. It uses a sequential learning process that sequentially changes the weak learner model, and the final prediction is determined by combining all model predictions from all iterations. Tianqi Chen claims that XGBoost has better performance because it has an overfitting control feature [34]. As time goes by, XGBoost has often become a champion in various data science competitions. Based on the explanation above, our system requires an algorithm that is able to classify accurately and quickly in real time. The suitable algorithm that meets our system requirement is XGBoost. Thus, we employ the XGBoost algorithm for classifying the breathing waveform in a real-time system.

## 4. Experimental and Analysis Results

The first part of this section provides selected parameters on the FMCW sensor. The second part describes the data collection and labeling. The last part is the experimental result and data analysis.

### 4.1. Experimental Setup

This study was carried out using an FMCW IWR 1443 mmWave radar platform from Texas Instruments (TI) [79] with a starting frequency of 77 GHz and a chirp frequency of 4 GHz. The chirp duration is 50 μs with the chirp rate 2 MHz and 250 samples per chirp. Each frame is configured to have two chirps. The details are shown in Table 1.

The experiments were conducted in a small room—3 × 3 m. The subject sat on a chair, and the radar was placed 1 m in front of the subject. The radar was positioned parallel to the chest at a height of about 1 m in the detectable area. The data was collected in binary format. We labeled these samples according to five different respiration classes. The participants were asked to imitate five breathing patterns. Observations were made on each subject with a duration ranging from 5 to 15 s for each class. During data recording, the subjects were not allowed to make any movement to reduce the random body movements that cause noise. The estimated frequency and amplitude will be better if the observation time is larger. However, the observation time is generally limited to the range of 5 to 15 s due to the inherent time-frequency sacrifice.

### 4.2. Data Collection and Labelling

In this study, we used the breathing waveforms as our data set. Through experiments, we collected 4000 breathing waveforms as the training and testing data. The system randomly divides the data set without following any rules into 80–20% train–test splits for experimental purposes. The collected breathing waveform consists of five classes: normal breathing, deep and quick breathing, deep breathing, quick breathing, and holding the breath.

Before training the data, the pre-processing step is necessary to normalize and eliminate the ambiguous and redundant data from the dataset. In data records, some pieces of data have missing values. To resolve the data, we removed data with missing values from the dataset. We cleaned the noise from the data for better performance and accuracy. The accuracy depends on the input data. We split the breathing waveforms into several data for every 5 s. For each data, we limit the window to 85 steps size. After pre-processing, finally, we had data set with details shown in Table 2.

The data set was used in the training process to train the classifier model. During the training process, the computer will learn and understand the data to obtain the expected model.

### 4.3. Experiment and Analysis Results

Before we conducted the experiment, the proposed method was implemented on hardware. The data was collected and labeled as the training datasets. To verify the accuracy of the proposed system, we conducted three experiments for detecting five respiration patterns. The first experiment was conducted without additional feature extraction. The second experiment was conducted using statistical feature extraction, and the third experiment was conducted using MFCC feature extraction.

The statistical feature extraction is used to identify the statistical character of data. In this study, the statistical features were derived from the statistical distribution of the respiratory signal data, such as the mean, median, maximum, variance, standard deviation, absolute deviation, kurtosis, and skewness.

- The mean is the average value of the population.
- The median or middle value is a measure of data centering. If the data is sorted, the observed value is in the middle.
- Maximum describes a greater value than or equal to all values in data.
- Variance presents a square of the average distance between each quantity and mean.
- Standard deviation is used to measure the amount of variation or dispersion of data. The standard deviation describes how far the sample deviates from the mean.
- Absolute deviation represents the absolute difference between each data point and the average. This explains the variability of the data set.
- Kurtosis defines the degree of “tailedness” of a distribution.
- Skewness is known as a measure of slope, which is a number that can indicate whether the curve shape is slanted or not.

Before we trained our data, we plotted it, which has been extracted using feature extraction, into a two-dimensional diagram of linear discriminant analysis (LDA) [94]. The aim is to see the effect of adding MFCC feature extraction. LDA is a classical statistical technique that can reduce the dimensions [94]. With LDA, we can also divide data into several groups (clustering) [94].

**Figure 9:**
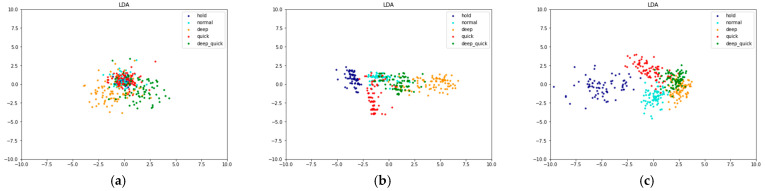
LDA data scattering point for (**a**) raw data, (**b**) data with statistic feature extraction, and (**c**) data with MFCC feature extraction.

Based on the LDA results, Figure 9 describes that MFCC makes the scattering point of one class to be closer and the scattering point for five different classes to be farther. Thus, it shows that MFCC feature extraction helps the classifier in clustering the data. As a comparison, we also show the effect of data extraction using statistical methods. We can see in Figure 9 that the scattering point of the data with the MFCC extraction feature has the least number of overlapping classes.

In the next step, the datasets were used to train the XGBoost model. After the entire training phase ends, the resulting model must be tested again using a test set. The evaluation/testing step aims to decide whether the model is good enough or not.

One of the problems in building a learning model is finding the optimal hyperparameter. For example, we need to decide the optimal batch size, the optimal epoch for running a deep-learning model, and the best optimizer for deep-learning models. Many other hyperparameters can be optimized, such as the dropout, number of nodes, number of layers, activation functions, and others. It is time-consuming to use trial and error, trying to change the parameters manually, one by one, to find the best model. One solution to this problem is to use GridSearchCV.

Grid search, as the name implies, looks for parameters in a given “grid”—for example, the number of epochs =—then, we need to decide which of the two values gives the best result. In this study, we used the following parameters:

- *n* estimators: [200 300 400], *n* estimators represent the number of sequential trees modelled in XGBoost.
- Max depth: [3 4 5], max depth means the maximum number of terminal nodes in a tree.
- Learning rate: [0.1, 0.01, 0.001], the learning rate is the learning parameters that control the change value in estimating the prediction. A smaller value causes a stronger model with specific characteristics of the tree. However, lower values will require a larger number of trees to model all relations and do a lot of computation.

The way the Grid Search works is by combining the values inputted in the hyperparameters. An example is when we want to find a combination of hyperparameters A = [1, 2] and B = [3, 4], then the Grid Search will look for all combinations of A and B, namely [1, 3], [1, 4], [2, 3], [2, 4] and choose the best combination based on the value of the highest CV Score. We found the best combinations to obtain higher accuracy. The process was carried out by brute force and reported which parameter has the best accuracy. As we have three parameters with three grids for each, we thus have 27 combinations.

CV, at the end of the word GridSearchCV, stands for cross-validation. This means that our input data will be divided by GridSearchCV into several folds to reduce the bias. In our study, we used five-fold cross-validation. It divided a set of samples randomly into five independent subsets, to do five repetitions for training and testing. For each test, a subset was left for testing and another subset for training. The degree of accuracy is calculated by dividing the total number of correct classifications by the sum of all instances in the initial data.

**Figure 10:**
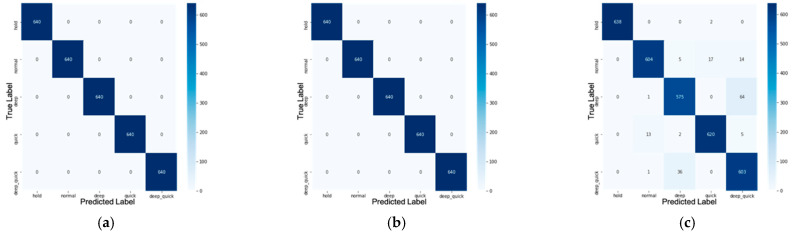
Confusion matrix for (**a**) raw data, (**b**) data with statistic feature extraction, and (**c**) data with MFCC feature extraction on training stage.

**Figure 11:**
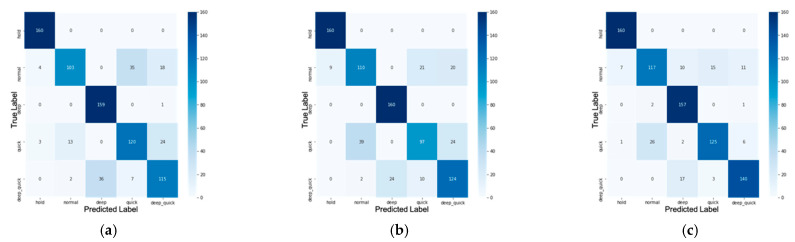
Confusion matrix for (**a**) raw data, (**b**) data with statistic feature extraction, and (**c**) data with MFCC feature extraction on testing stage.

XGBoost model performance is calculated through a confusion matrix. The confusion matrix presents the amount of data classified correctly and incorrectly. The effectiveness and performance of a machine learning model can be measured by calculating its accuracy. Finally, the result is shown in Figure 10, Figure 11 and Table 3.

In the confusion matrix, most misclassifications come from predicting deep quick breathing to be deep breathing, normal breathing to be quick breathing, and vice versa. A possible reason is that these two classes have almost the same pattern but are different in the depth of breathing, shown in the amplitude of the waveform. This might happen because the amplitude of the respiratory signal is sensitive to the time window in the normalization process. Besides, the accuracy of the model depends on the input data, whereas chest displacement waves have different variations depending on several factors such as the state of health, location of measurement, variation between people, etc.

Based on the experiments above, we showed that adding MFCC feature extraction gives a better result than without and with statistical feature extraction. Thus, we implemented our proposed system in real time by using MFCC feature extraction. 

Let us define X as a disease name. Then, we have four definitions as follows.

From Table 4, there is one important case that needs special attention—false positive. When the system does not detect the patient′s disease, in reality the patient is suffering from the disease. This is very dangerous. For example, if the patient has COVID-19 but the system detects that the patient′s condition is normal, then the patient will not immediately receive the right treatment. On the other hand, true negatives and false negatives also need attention. If the system detects that a patient is suffering from disease A, but in reality, the patient is suffering from disease B, then the patient will not receive the right treatment. However, if the system detects that the patient suffers from X disease, but the patient is normal, the condition is not dangerous.

Precision is defined as $Precision=\frac{TP}{TP+FP}$. High precision shows that the class can be classified well or have a low FP. Recall is defined as $Recall=\frac{TP}{TP+FN}$. High recall indicates that the class has a low FN. The f1-score is the average of precision and recall that takes these two metrics into $f1\;score=2\;\frac{precision\;*\;recall}{precision\;+\;recall}$. From the confusion matrix in Figure 11, we thus have the classification report shown in Table 5.

As mentioned before, since a false-positive result is the most dangerous condition, we need to achieve a better precision than recall. To detect deep quick, and quick class, XGBoost with MFCC feature extraction achieves the best precision. However, for the deep class, XGBoost with statistic feature extraction gives the best precision. For the normal and hold class, XGBoost without feature extraction has the best precision.

Patients with COVID-19 usually have a quick and short breath at unexpected times. This condition is related to class 4, quick breathing, or short breathing. Thus, if we need to detect patient with COVID-19, it is better to use XGBoost with MFCC feature extraction because it achieves the best precision in detecting quick/short breathing.

We ran the system into a real-time experiment. We conducted five measurements with an object located approximately 1 m in front of the sensor. The results for the detection and classification of breathing waveforms in real time can be seen in Table 6. Table 6 shows the estimated range of the target, chest displacement waveform, estimated breathing rate, and breathing waveform. Five figures in the first left column are the azimuth heat map that shows the range and angle estimation for the object in front of the sensor. It illustrated that the sensor detects 0.1 to 0.5 Hz vibration at approximately 1 m. The figure in the next column shows real-time chest displacement, and the figure in the right column is a real-time breathing waveform. 

To clarify whether the breathing waveform was accurate or not, we tried to estimate the respiration rate calculation. The estimated value of the respiration rate was then compared with counting the breathing rate manually. The respiration rate calculation was performed by counting the number of inhalation and exhalation cycles in one minute. The result of the breathing rate is shown in the last two columns of Table 6.

The first experiment was detecting the normal breath, shown in the first row in Table 6. The results show us that the object was detected at about 1.20 m with an angle of 30 degrees. The breathing waveform has a constant breathing waveform and similar pattern during the time. The estimated breathing rate was 20.51 breaths/min.

The second measurement was detecting the deep quick breath, shown in the second row in Table 6. The object was detected at the range of 1.23 m and 30 degrees from the sensor with a breathing rate of 23.44 breath/min. The breathing waveform presents a big amplitude with a higher frequency (higher respiration rate) compared to the normal breathing rate.

The third observation was conducted for a deep breath, shown in the third row in Table 6. The vibration was detected at 1.17 m from the sensor. The detected breathing rate was 17.58 breaths/min. Deep breathing waveform shows a big and large amplitude with a lower respiration rate compared to normal breath.

The fourth experiment detected the quick breath, shown in the fourth row in Table 6. The breathing waveform was detected at 1.88 m from the sensor with a small amplitude and high frequency (high respiration rate). The detected breathing rate was 23.51 breaths/minute.

The last experiment measured the holding breath class. The results show us that the object was detected at about 1.08 m with an angle of 30 degrees. The breathing waveform is almost disappeared, and the amplitudes are close to zero. The estimated breathing rate was 0 breaths/min.

Based on our real-time experiment, Table 6 presents that our real-time implementation can successfully classify five different breathing waveform classes. This shows us that the proposed system can be used to monitor and classify the breathing waveform in real-time. Besides, the breathing rate result shows that our respiration rate has a close value to the manual calculation of the breathing rate, as shown in Table 6.

## 5. Conclusions

In this paper, we have proposed a non-contact monitoring and classification system for breathing patterns using XGBoost classifier and MFCC feature extraction. Based on the results, the system reached 87.375% accuracy. We also compared the impact of adding MFCC feature extraction to statistical feature extraction and without feature extraction. The results show that the XGBoost classifier with the MFCC feature extraction achieves the best accuracy in classifying five breathing patterns. Thus, we implemented our proposed system in real time by using MFCC feature extraction. Our real-time experiment verifies that our system successfully classifies five different classes of breathing waveform. This shows us that the proposed system can be used to monitor and classify the breathing waveform disorder in real time.

The proposed system will not be a perfect substitute for a professional doctor. It is hoped that this assistance will help practitioners to monitor and analyze the patients. In some cases, the practitioner may make mistakes, pay little attention to the patients, or perform poor report analyses. Thus, it will act as a better solution for now.

In the future, more breathing patterns and classification algorithms will be investigated, and a larger data set will be built. It is hoped that the detection of multiple subjects can be carried out, and the classification model can also be optimized. Since this sensor can be connected to the computer, it also allows us to monitor the breathing waveform with a centralized system. Hence, the supervision of breathing patterns with a centralized system can be developed. In addition, FMCW can also be used to conduct measurements behind interrupted objects such as curtains, walls and others. Therefore, the development of this study is not only useful for the medical field but also for other fields that require detection without physical contact, such as searching for and locating people trapped under rubble. Thus, it would be very helpful for saving lives during a disaster.

Under a controlled environment, all the mentioned methods can work properly. However, monitoring and measuring the breathing pattern in a noisy environment is a challenge that needs to be overcome to make the system stronger and more reliable in the future. 

## Figures and Tables

**Table 1:** Radar parameter setting.

Starting Frequency	Bandwidth	Chirp Rate	Samples Per-Chirp	Chirps Per-Frame	Chirp Duration	Frame Duration	Range Resolution	Max Unambiguous Range
77 GHz	4 GHz	2 MHz	250 samples	2	50 μs	50 ms	0.0375	9 m

**Table 2:** Data set for training and testing.

Class	Training Samples	Testing Samples
Normal breathing	640	160
Deep and quick breathing	640	160
Deep breathing	640	160
Quick breathing	640	160
Holding the breath	640	160
Total	3200	800

**Table 3:** Training and testing accuracy for the raw data set, data set with statistic feature extraction, and with MFCC feature extraction.

Feature Extraction	Training Accuracy	Testing Accuracy
without feature extraction (raw data)	100%	82.125%
statistic	100%	81.375%
MFCC	95%	87.375%

**Table 4:** Confusion matrix 2×2.

True Positive (TP)	True Negative (TN)
Prediction: the system detects that the patient suffers from X disease Reality: the patient suffers from X disease	Prediction: the system detects that the patient suffers from X disease Reality: the patient does not suffer from X disease
**False-Positive (FP)**	**False-Negative (FN)**
Prediction: the system does not detect that the patient suffers from X disease Reality: the patient suffers from X disease	Prediction: the system does not detect that the patient suffers from X disease Reality: the patient does not suffer from X disease

**Table 5:** Classification report for confusion matrix in Figure 11.

Class.	Raw (without Feature Extraction)			With Statistic Feature Extraction			With MFCC Feature Extraction		
	Precision	Recall	f1-Score	Precision	Recall	f1-Score	Precision	Recall	f1-Score
Normal	0.873	0.644	0.741	0.728	0.688	0.707	0.807	0.731	0.767
Deep quick	0.728	0.719	0.723	0.738	0.775	0.756	0.886	0.875	0.881
Deep	0.815	0.994	0.9	0.87	1	0.93	0.844	0.981	0.908
Quick	0.741	0.75	0.745	0.758	0.606	0.674	0.874	0.781	0.825
Hold	0.958	1	0.979	0.947	1	0.973	0.952	1	0.976

**Table 6:** Real-time measurement using TI-IWR 1443 for five breathing classes.

Class	Real-Time Measurement	Breathing Rate	
		Manual	Measured
Normal	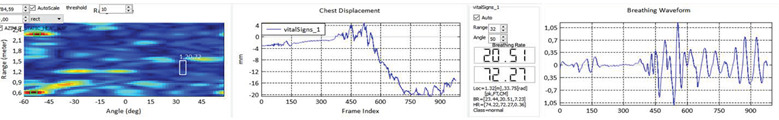	21	20.51
Deep Quick	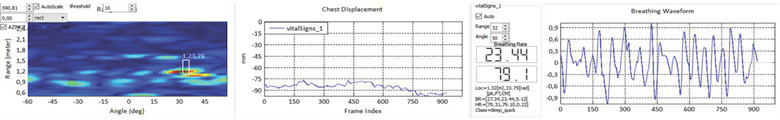	23	23.44
Deep	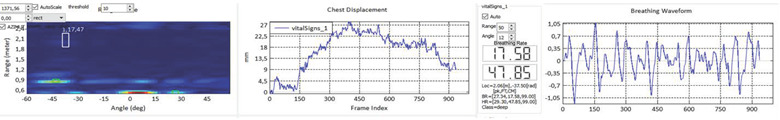	17	17.58
Quick	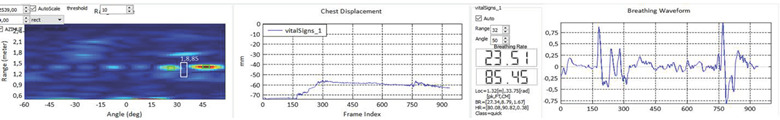	22	23.51
Hold	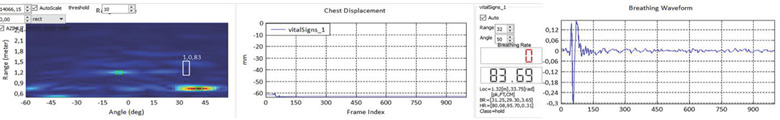	0	0

## Data Availability

Not applicable.
